# Reform in Expert Medical Testimony

**Published:** 1897-11

**Authors:** 


A Monthly Review of Medicine and Surgery.

EDITORS:

THOMAS LOTHROP, M. D. - - WM. WARREN POTTER, M. D.

All communications, whether of a literary or business nature, should be addressed to the managing editor: 284 FRANKLIN STREET, BUFFALO, N. Y.

VOL. XXXVII. NOVEMBER, 1897. No. 4.

### REFORM IN EXPERT MEDICAL TESTIMONY.

FOR some time past there has been a feeling among many members of both the legal and medical professions, that the system in vogue in the courts relating to expert medical testimony is inadequate to meet the necessities of justice and often unfair to prosecution or defense—one or both. Recent celebrated cases tried in the courts, notably one that occurred in Batavia during the past summer, have served to stimulate renewed inquiry into the propriety of reforming the methods of practice by seeking amendments to the statutes through the legislature.

At the recent meeting of the Medical Association of Central New York, held at Buffalo, this subject was debated with intelligence by a number of physicians from a medical point of view, and by Tracy C. Becker, Esq., on the part of the legal profession. The subject was introduced through the report of a committee that was appointed last year. The report recommended a scheme for certain changes in the statutes, so as to require all medical testimony to be made in the form of a medical commission, which should investigate the case at issue and which should be paid by the state, not by either defendant or prosecutor. Dr. A. W. Henckell, of Rochester, followed the chairman of the committee and adduced some forcible arguments in favor of the course recommended by him and his colleagues, giving many instances in which the ends of justice had not been aided—even thwarted—by the present method of calling upon paid expert testimony. Mr. Tracy C. Becker, of Buffalo, went into the legal aspects of the present and proposed limitations of expert testimony and showed that many justices of the supreme court and eminent lawyers do not consider that expert testimony should be allowed to rest in its present position. A reprint of all testimony in the possession of the association on this matter will be put into pamphlet form and distributed soon ; in the meantime the subject remains in the hands of the committee, which has merely reported progress.

It is to be hoped that the agitation of this subject, in such an intelligent form, will lead to the correction of abuses that grow out of the chaotic methods under present practices. Any man who has ever been a medical expert witness in an important trial at bar will appreciate the propriety of being relieved from that kind of embarrassing, not to say improper, cross-examination that a designing lawyer not trained to the gentlemanly courtesies of polite society may subject a witness.

One of the most delightful memories of the late Mr. Laning, who possessed many charming qualities of head and heart, was that he was always equally polite in the cross-examination of an expert witness as he was in the direct ; in other words, whether the witness was his own or his opponent's he always found courteous treatment at the hands of this distinguished lawyer. Mr. Laning never forgot that he was a gentleman even when subjecting a witness to a searching examination, which he was always sure to do if occasion demanded it. Were all lawyers like Mr. Laning perhaps the present system might not be as unjust in its application to trial cases.

That some change from the present system is needed all who have any knowledge of the subject are doubtless prepared to admit. The only question at issue relates to the method that should be adopted in its stead. A commission to be appointed by the court and paid by the state, seems to be the favorite plan with the majority who have expressed an opinion on the subject. Another proposition is to authorise a commission of three—the court, prosecution and defense each to appoint a member. In any event, the report of a commission, it seems to us, should be made in writing and be conclusive on the subject-matter with which it deals. At any rate, some plan should be devised to take out of the hands of the lawyers the opportunity to so cross-examine an expert witness as to apparently make him stultify himself or neutralise the effect of his testimony.

Most trials at law as now conducted seem to be adroit schemes for obtaining the miscarriage of justice. Even in civil actions the delays and perplexities are such that the victor is often worse beaten than the vanquished. At the present day only the very rich or the very poor can afford the luxury, satisfaction or entertainment of a lawsuit. Wealthy persons can afford to squander a few hundreds on the chances of a verdict as a mere pastime, while the very poor find an occupation and sometimes a competency in the uncertain turn of the ballots in a jury box. The only proper course for a medical expert witness to pursue under the present system is to charge a fee sufficient to adequately compensate him for all the delays and annoyances to which he will assuredly be subjected.

It is to be hoped, however, that the agitation of this subject will continue until medical expert testimony shall be established on a basis creditable alike to the science of medicine and the art of law. The report of the committee of the Medical Association of Central New York, to be made at Auburn next year, will be looked forward to with much interest by all concerned in this much-needed reform.

